# Mapping the force field of a hydrogen-bonded assembly

**DOI:** 10.1038/ncomms4931

**Published:** 2014-05-30

**Authors:** A. M. Sweetman, S. P. Jarvis, Hongqian Sang, I. Lekkas, P. Rahe, Yu Wang, Jianbo Wang, N.R. Champness, L. Kantorovich, P. Moriarty

**Affiliations:** 1 School of Physics & Astronomy, University of Nottingham, Nottingham NG7 2RD, UK; 2 School of Physics and Technology, Centre for Electron Microscopy and MOE Key Laboratory of Artificial Micro- and Nano-structures, Wuhan University, Wuhan 430072, China; 3 Department of Physics, King’s College London, The Strand, London WC2R 2LS, UK; 4 Department of Physics and Astronomy, University of Utah, Salt Lake City, Utah 84112-0830, USA; 5 School of Chemistry, University of Nottingham, Nottingham NG7 2RD, UK; 6 These authors contributed equally to this work

## Abstract

Hydrogen bonding underpins the properties of a vast array of systems spanning a wide variety of scientific fields. From the elegance of base pair interactions in DNA to the symmetry of extended supramolecular assemblies, hydrogen bonds play an essential role in directing intermolecular forces. Yet fundamental aspects of the hydrogen bond continue to be vigorously debated. Here we use dynamic force microscopy (DFM) to quantitatively map the tip-sample force field for naphthalene tetracarboxylic diimide molecules hydrogen-bonded in two-dimensional assemblies. A comparison of experimental images and force spectra with their simulated counterparts shows that intermolecular contrast arises from repulsive tip-sample interactions whose interpretation can be aided via an examination of charge density depletion across the molecular system. Interpreting DFM images of hydrogen-bonded systems therefore necessitates detailed consideration of the coupled tip-molecule system: analyses based on intermolecular charge density in the absence of the tip fail to capture the essential physical chemistry underpinning the imaging mechanism.

## Abstract (web summary)

Hydrogen bonding is an important force in nature, directing and controlling the assembly of numerous natural and synthetic structures. Here the authors show that the interpretation of intermolecular contrast in atomic force microscope images of hydrogen-bonded assemblies necessitates detailed consideration of the coupled tip-molecule system.

## Graphical abstract

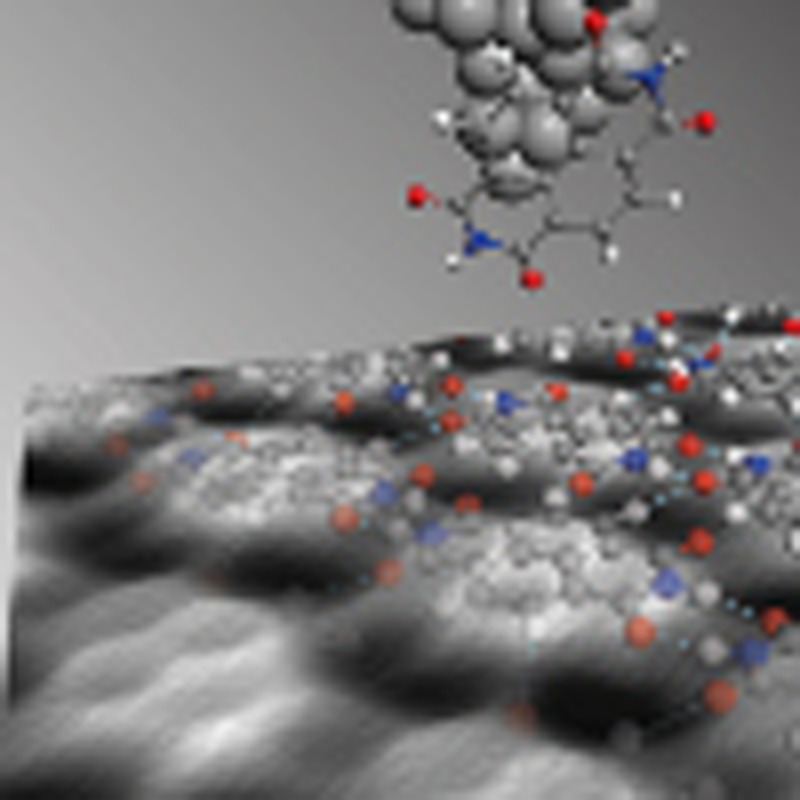

If the concept of the chemical bond is indeed a “convenient fiction”^1^ with any attempt at a rigorous definition “bound to be impoverishing”^2^, then hydrogen bonds represent a particularly compelling example of just how nebulous the classification of chemical interactions can be. Despite almost a century of study^3^, the IUPAC definition of the hydrogen bond was changed^4^ as recently as 2011, reflecting the remarkable complexity of the interaction^5^ and the difficulty of delineating the various physicochemical components—including electrostatic, dispersion, covalency, and exchange contributions—of hydrogen bonding.

The amount of literature on hydrogen bonding is, unsurprisingly, voluminous, given its central importance in so many scientific fields, including biochemistry and biomaterials^6^, organic synthesis^7^, and, of particular relevance to this paper, crystal engineering and self-assembly at surfaces^8^. With regard to the latter area of research, there has been a particularly intense focus of late on the exploitation of hydrogen bonding (and a variety of both non-covalent interactions and covalent bonds) as a means to engineer supramolecular lattices and templates at surfaces^9^,^10^,^11^. Scanning tunnelling microscopy (STM) has been used to provide molecular resolution imaging of those systems but traditional STM is sensitive only to the density of states within the energy window defined by the tip-sample bias voltage, necessarily limiting the spatial resolution achievable^12^.

Dynamic force microscopy (DFM, also known as non-contact atomic force microscopy) is not subject to this limitation because, as highlighted by Giessibl^12^, it has the potential to probe the total-electron density (TED). With a suitably functionalized probe, and operation in the regime of the tip-sample potential where the repulsive force makes a strong contribution, remarkably high resolution can be attained, enabling the visualization of not only intramolecular structure^13^, but, as shown very recently, features which have been identified as *inter*molecular hydrogen-bonds^14^.

Intriguingly, and before the publication of the DFM results, it had been discovered that the very high spatial resolution attained in DFM could also be achieved in STM by similar functionalisation of the tip and operation at small tip-sample separations 15,16,17,18. This variant of STM, scanning tunnelling hydrogen microscopy (STHM), has also produced remarkable images of organic monolayers, which exhibit striking intermolecular contrast interpreted as being derived from hydrogen-bonds (although the detailed contrast mechanism is not currently understood)^17^. In STHM, the experimental observable is, however, the junction conductance and, particularly for small tip-sample separations, there is a complex relationship between this observable and the tip-sample force.

In contrast, for DFM the tip-sample force can be extracted in a relatively straight-forward manner from the frequency shift experienced by the oscillating sensor. As we discuss here, this allows quantitative measurements of the molecular force field (and potential energy landscape) for hydrogen-bonded molecules to be attained with a tip terminated by a single molecule (the ‘sensor’ molecule). Note that, as described below, tip functionalization in our case occurs via routine scanning, rather than through the tip modification strategies applied to date to achieve intra^13^,^19^ and intermolecular^14^ contrast in DFM. We find that intermolecular contrast appears only when the short-range tip-sample force gradient, 
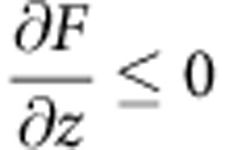
; any long-ranged attractive electrostatic contribution to the maxima observed at the expected hydrogen-bond positions is not detectable within the accuracy of our measurements. Moreover, while it is clear that relaxation of the tip can play an exceptionally important role in the generation of both intramolecular^19^ and intermolecular^20^ features, we demonstrate that even when the atomic coordinates and the electronic density of the tip-sample system are ‘frozen’ in place, theoretical calculations reproduce the intermolecular features observed in the experiment.

The central result we report here is that intermolecular contrast in DFM can only be understood in the context of the coupled tip-sample system: the fundamental mechanism underpinning the observation of intermolecular features in DFM images is the repulsion present between the tip apex and sample. We show that consideration of the TED alone is insufficient to explain the contrast and that key insights into the origin of intermolecular contrast may be obtained by considering the fraction of charge redistributed in the bond region owing to the tip-sample interaction as a function of the approach of the probe.

## Results

### Intra and intermolecular contrast in a hydrogen-bonded assembly

**Figure 1:**
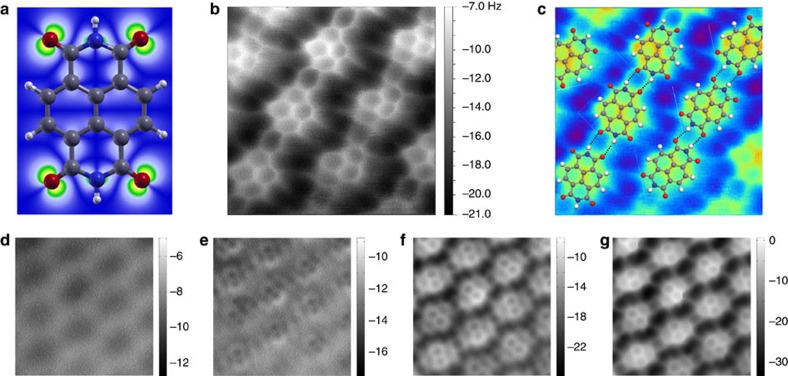
Intermolecular contrast in a 2D hydrogen-bonded assembly. (**a**) Ball-and-stick model of NTCDI with a partial charge density isosurface superimposed (Colour coding: grey, carbon; white, hydrogen; red, oxygen; blue, nitrogen); (**b**) Constant height DFM image of a hydrogen-bonded NTCDI island on the Ag:Si(111)- (√3 x √3) R30° surface acquired at 77 K. Image size 2.1 by 2.0 nm (oscillation amplitude, 275 pm); (**c**) Overlay of the model of NTCDI on a contrast-adjusted section of the image shown in **b**. The primary intermolecular H bonds (N-H˙˙˙O) are shown as black lines. (**d**–**g**) Series of DFM images of a different NTCDI island taken at 5 K with progressively smaller tip-sample separations and an oscillation amplitude of 110 pm. Tip-sample separations, (i.e. Δ)z, relative to that associated with the Δf setpoint used for atom tracking (that is, Δz=0) are (**d**) −17 pm, (**e**) −67pm, (**f**) −125 pm and (**g**) −175 pm. Image size 3.3 by 3.3 nm.

The molecule chosen for our DFM studies of hydrogen bonding is naphthalene tetracarboxylic diimide (NTCDI, Fig. 1a), which has previously been shown to form hydrogen-bonded chains and two-dimensional (2D) assemblies on the Ag:Si(111)-(√3 × √3) R30° surface^21^,^22^, also used in our work. (From this point onwards we shall refer to this surface as Ag-√3). Figure 1b is a constant height DFM image of a NTCDI assembly, attained using a measurement protocol similar to that introduced by Gross *et al.*^13^ There are a number of noteworthy features in this image. First, both well-resolved intramolecular contrast and clear maxima at the expected positions of the intermolecular hydrogen-bonds are observed. The latter is rather similar to that previously observed by Zhang *et al.*^14^ in DFM images of 8-hydroxyquinoline (8-hq) molecular assemblies on Cu(111), and by Kichin *et al.*^16^ in STHM images of 3,4,9,10-perylene-tetracarboxylic-dianhydride (PTCDA) monolayers adsorbed on Au(111). In Fig. 1c we have overlaid a schematic model of the primary H-bonding interaction (N-H˙˙˙O) between NTCDI molecules on a contrast-adjusted version of Fig. 1b to highlight the correlation between the intermolecular contrast observed in the DFM image and the expected positions of the hydrogen bonds (also see Supplementary Fig. 1 for STM and constant Δf images). Weaker inter-row contrast at the expected positions for C-H˙˙˙O hydrogen bonds is also often observed, and is particularly clear in Fig. 1g, for example. As we highlight in Supplementary Figs 2–4, however, it is not always straight-forward to distinguish intermolecular bonding from features which arise from the close proximity of two molecules. A second key feature of the DFM images in Fig. 1 is that no deliberate functionalization of the apex of the probe was carried out before their acquisition. Nonetheless, images of this type are routinely acquired owing, we believe, to the ease with which NTCDI molecules are transferred from the surface to the tip apex. (See the Supplementary Methods and discussion below for more details). It is worth noting here that some of our images were acquired at 77 K (such as Fig. 1b), despite 5K temperatures exclusively being used hitherto to acquire intramolecular contrast. We stress that hydrogen-bond-derived intermolecular features are observed only at tip-sample separations where strong intramolecular contrast is also resolved. Figure 1d–g show the evolution of contrast in the NTCDI assembly as a function of tip-sample separation.

### Mapping tip-sample forces

**Figure 2:**
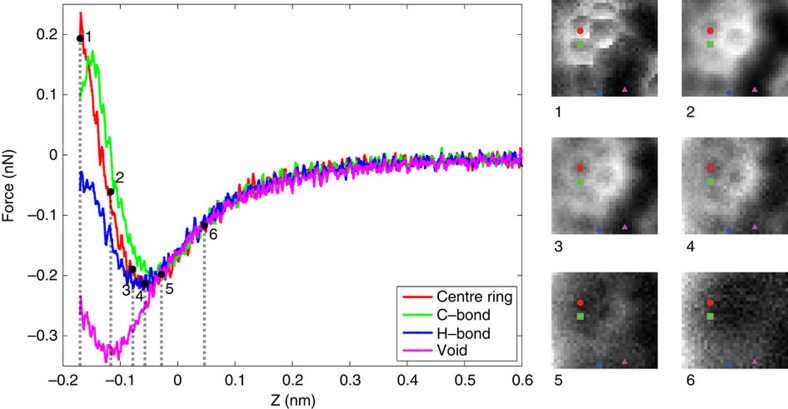
Quantifying the short-range forces responsible for intermolecular contrast. Short-range force curves for a H-bonded NTCDI molecule in a 2D assembly, which were acquired at the centre of a carbon ring, above a C–C bond, above a hydrogen bond region, and in a void region between the molecules are plotted in red, green, blue and purple, respectively. The curves were extracted from a spectroscopy grid acquired at 5 K. Slices through the spectroscopy grid acquired at the *z* values labelled as 1–6 on the force curves are shown as images 1–6 to the right. Note, in particular, the absence of intra- and intermolecular contrast in the region of the force curve where the force gradient is positive. Distinct intermolecular contrast appears only when the force gradient becomes negative, that is, when the repulsive component of the tip-sample interaction is sufficiently large to cause the force gradient to change sign. The oscillation amplitude here was 110 pm.

To quantify and map the forces and energies underpinning the intermolecular contrast observed in Fig. 1 we have used atom-tracking-enabled three-dimensional force spectroscopy^23^. Figure 2 shows the short-range force curves extracted from the spectroscopic matrix at various intra- and intermolecular positions. (See Supplementary Discussion, Supplementary Fig. 5 for the associated potential energy landscapes and Supplementary Movies 1 and 2 showing successive slices through the three-dimensional maps). It is only when the repulsive component of the tip-sample potential is sufficiently large to cause the force gradient to change sign from positive to negative that we see clear intermolecular (and intramolecular) contrast. This is a strong evidence suggesting that intermolecular contrast for the NTCDI system cannot arise from the relatively long-ranged electrostatic component of the hydrogen-bonding interaction. Figure 2 also demonstrates that there are only very subtle differences in the force curves measured above the C–C bonds in the NTCDI molecule, in the hollow sites at the centre of the carbon rings and in the region of the hydrogen-bond (see also Supplementary Figs 6–9 for additional force-maps and intermolecular separation measurements).

### Identifying the tip termination

Thus far, we have argued that our images arise from a NTCDI molecule terminating the tip, in analogy with the CO termination described by Gross *et al.*^13^ For a detailed quantitative analysis of the experimental data, and to elucidate the physicochemical mechanisms underpinning the intermolecular contrast seen in Figs 1 and 2, it is essential to first know the orientation of the tip-adsorbed NTCDI molecule—an experimental ‘unobservable’ which we determine here by direct comparison of the measured short-range force curves with the results of density functional theory (DFT) calculations involving a variety of plausible tip types.

**Figure 3:**
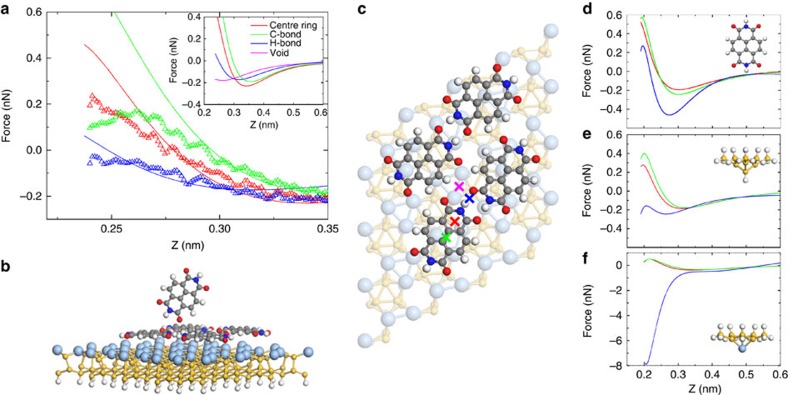
Comparison of experimental and calculated tip-sample interactions. (**a**) Calculated (−) and measured (Δ) force curves overlaid in the negative force gradient region of the tip-molecule interaction. Inset: complete calculated F(*z*) curves, including the data for the bare surface position (that is, **X** in Fig. 3c) whose minimum lies at a much smaller tip-sample separation. (**b**) Ball-and-stick model of the full simulated system with an O-down oriented NTCDI tip (side-on view). (**c**) Top-down schematic of the simulated NTCDI island with spectral sites marked as crosses. (**d**–**f**) Calculated *F(z)* curves for alternative tip structures: H-down NTCDI, H-terminated and an Ag-terminated silicon cluster, respectively.

The complete tip-NTCDI island-(Ag-√3) system was simulated using an *ab initio* DFT method via the CP2K code (see Methods and the schematic diagrams in Fig. 3). To calculate *F*(*z*) curves for comparison with the experimental short-range force-distance spectra we positioned the simulated tip at an initial height of 0.6 nm with respect to the surface molecule at several sites marked in Fig 3c. The tip-sample separation was then reduced in small quasi-static steps of 0.1 Å towards the surface, relaxing the system geometry at each point. The calculated total energies were then fitted with a curve comprising a summation of inverse power laws and analytically differentiated to obtain the tip-sample force (see Supplementary Fig. 10).

To determine which of the simulated tip structures provided the best agreement with experiment we compared 
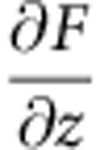
 (in the negative force gradient regime) and the maximum attractive force with those for the measured short-range curves. We found that a NTCDI-terminated tip in the O-down orientation (Fig. 3b) provided the best agreement out of all of the tip apices we tested, particularly in the negative force gradient region, as shown in Fig. 3a. Although the alternative tips we considered also demonstrated good agreement with experimental force spectra acquired above the C–C and carbon ring positions (suggesting all would be capable of intramolecular resolution), the calculated maximum attractive force for the intermolecular H-bond location was generally overestimated, or failed to become sufficiently repulsive (Fig. 3d–f).

### TED vs density depletion

Following a similar approach to earlier work^14^ we calculated the TED for the simulated NTCDI island adsorbed on the Ag-√3 surface. Although a previous report claimed good agreement between the TED and constant height DFM images of intermolecular bonds^14^, we find that the experimental contrast in the regions associated with H-bonding in the NTCDI assembly was not at all reproduced well. Examination of the TED showed that the electronic density in the region of H-bonding was over an order of magnitude (~0.08 versus ~1.5 *e*/*a*_0_^3^) smaller than that over the C–C bond regions (Supplementary Fig 11). In experiment, as Figs 1 and 2 clearly show, we do not observe this striking difference in contrast between the intra and intermolecular features.

It is therefore clear that a consideration of the TED alone is insufficient to explain the observation of strong intermolecular contrast in our DFM images. Importantly we observe good agreement between the measured and calculated *F*(*z*) data beyond the force turnaround in our full DFT calculations, suggesting that consideration of the tip-sample interaction is vital to understand the observed contrast. To examine the role of the variation in charge density owing to tip-sample interactions we calculated both the TED and the electron density difference (EDD). The EDD was obtained by first calculating the TED for the isolated surface (that is, the Ag-√3 slab along with the NTCDI molecular island) and also the isolated NTCDI tip at each step of the *F*(*z*) calculation. These two densities were then summed together and subtracted from the relaxed total density for the full system. Therefore, the remaining subtracted EDD describes the interaction caused by the presence of the tip. This allows us to examine the tip’s effect on the simulated system by determining the fraction of charge redistributed owing to the interaction of the probe and target molecule.

**Figure 4:**
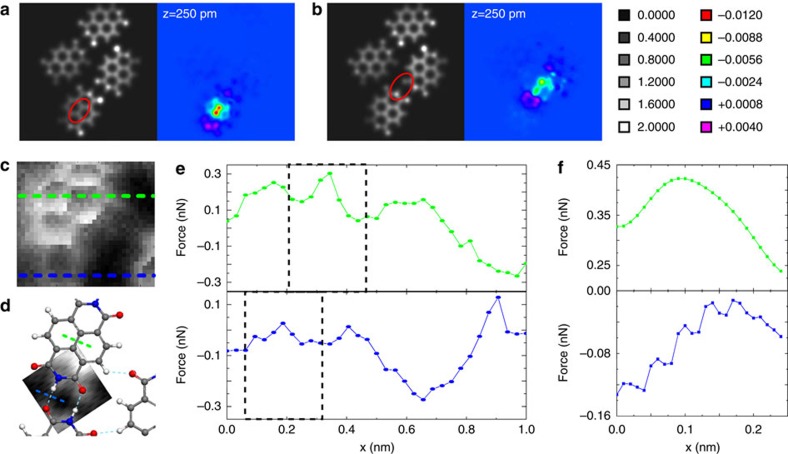
Relaxed and frozen electron densities. (**a**) Calculated TED (left) and EDD (right) plots for an O-down NTCDI tip at the C–C location of a networked NTCDI at a tip-sample separation of *z*=250 pm (corresponding to *z* value #1 in Fig. 2); (**b**) Equivalent plot for the H-bond location. Note comparable intensity of EDD features in **a** and **b**. (Density scale represented in units of electrons per bohr^3^). EDD plotted at 50 pm above molecular plane. (**c**) Slice through the three-dimensional force field of Fig. 2, also corresponding to *z* value #1 in that figure. The green and blue lines indicate the positions of the line profiles for the C–C bond region, and hydrogen-bond region which are shown in **e**; (**d**) Simulated image of hydrogen-bond region generated by fixing all atomic coordinates and the electron density of the tip and sample. This slice was taken from a simulated three-dimensional force field and was selected on the basis of a best match to the experimentally measured forces. The dotted lines through the C–C bond (green in (**c**)) and the intramolecular bond region (blue in (**c**)) regions represent the positions of the simulated line profiles shown in **f**. (**e**) Upper: experimentally measured force profile across an intramolecular C–C bond. Lower: experimental force profile for the hydrogen bond region. (**f**) Line profiles through the simulated three-dimensional force field, at the H-bond and C–C bond positions. Compare the experimental profiles in **e**. Note that the small oscillation of the force observed for the simulated hydrogen-bond region arises from a numerical artefact owing to the finite self-consistent field (SCF) grid size used to calculate the density. (See the Supplementary Information file for more information).

Both the TED and EDD were plotted as x-y slices positioned at 50 pm above the NTCDI molecular plane in Fig. 4. This position was chosen to intersect the EDD in the region of highest density. (For comparison, slices at 100 pm are shown in Supplementary Fig. 12). To quantify the effect of the molecular probe on the electronic density we calculated the TED and EDD with the O-down NTCDI tip at each tip-sample separation from the calculated *F*(*z*) curves. The same lateral positions as the force spectra in Fig. 3 were chosen to facilitate a comparison with our experimental data. Figure 4a,b are the TED and EDD maps above a NTCDI C–C bond, and an intermolecular H-bond, respectively, at a tip-sample separation corresponding to Position 1 in Fig. 2. Images at the other locations are shown in Supplementary Fig. 13.

## Discussion

The most striking observation we make in Fig. 4 is that while the TED provides little evidence of the intermolecular features seen in the experiment, the EDD shows remarkably similar depletion localized above the C–C and hydrogen-bonds. At *z*=250 pm in particular, where the intramolecular contrast is strongest, we observe a region of depletion with similar magnitude over each bond type (H-bond, −0.009 e/a_0_^3^; C bond, −0.012 e/a_0_^3.^).

The charge density depletion driven by the intermolecular force at the tip-sample junction highlights the amount of the charge density that is affected by the repulsive interaction. It is clear that only a fraction of the TED is responsible for the repulsive interaction (recall, from Fig. 2 that the short-range force curve is overall attractive at tip-sample separations where intermolecular features are observed). By comparing the magnitude of the TED and the EDD in the C–C and H-bond locations we find that only ~0.8% of the charge is affected in the C–C region whereas a much larger fraction, ~11% is affected in the H-bond location. As such, it appears that only a certain amount of charge from each location is depleted owing to the repulsive tip-sample interaction. Therefore, contrast over the H-bonding region is effectively accentuated compared with that related to the bonds within the molecule itself, in a much stronger manner than a map of the TED might imply. This tip-induced accentuation is the key for explaining the comparable intensity of the intramolecular and intermolecular features in the experimental DFM images.

Gross *et al.*^19^ first showed that the relaxation of the ‘sensor’ molecule adsorbed at the tip apex—in their case, CO—plays an important role in the image formation mechanism for intramolecular bonds, leading to a substantial increase in apparent bond length. More recently, it has been demonstrated that relaxation of the tip apex can also generate apparent intermolecular contrast^20^ owing to the low value of the torsional spring constant for a CO-terminated apex^24^,^25^. We have therefore paid particular attention to the possibility that displacement and ‘flexure’ of the NTCDI molecule at the tip apex may contribute to image contrast. Supplementary Fig. 14 shows that displacement of the O-down NTCDI molecule is minimal at the typical tip-sample forces at which we observe intermolecular (and intramolecular) contrast. Nonetheless, the overall behaviour of the O-down NTCDI-terminated tip apex is broadly comparable to that of a CO termination (Supplementary Fig. 15). To fully elucidate the contribution of tip mechanics to the observation of intermolecular features we have therefore simulated the force field for the hydrogen bond and C–C bond regions of the NTCDI assembly, as probed using an O-down NTCDI tip apex where neither the tip nor the sample are permitted to relax in any way. That is, the atomic coordinates and the electronic density of the simulated system were ‘frozen’ in place and, thus, only the electronic density of the unrelaxed system contributes to the tip-sample force. We note that even though the effect of electronic redistribution is absent in the frozen calculations the force curves show similar overall trends to the fully relaxed case (Supplementary Fig. 16; full details of the calculation are given in the Supplementary Methods).

Figure 4c,d show 2D slices through the experimental and the simulated force fields respectively, for a tip-sample separation which best matches the experimentally measured and calculated forces. A comparison of the line profiles through the hydrogen-bond and C–C bond regions from the experiment (Fig. 4e) and from the ‘frozen’ geometry/density calculation (Fig. 4f) indicates that intermolecular contrast at the hydrogen bond position is observed even when no relaxation of the system is permitted. This indicates that while tip relaxation is an important contribution to the generation of contrast in DFM images^19^,^20^,^25^, it need not be the only mechanism underpinning the observation of intermolecular features. Our somewhat crude—though nonetheless computationally demanding—calculation using fixed geometry and density also provides reasonable quantitative agreement with the experimental data: the magnitude of the variations of the tip-sample force along the H-bond and C–C bond features shown in Fig. 4e,f are roughly comparable. The ‘pinning’ of the atomic geometry and electronic density of course misses essential aspects of the physics, including the relaxation giving rise to the maxima seen in Fig. 4a,b, and thus could not be expected to provide precise quantitative agreement with experiment (see also Supplementary Fig. 17).

In summary, the observation of clear contrast maxima at the expected positions of intermolecular hydrogen bonds in a 2D molecular assembly originates from electron repulsion induced at the tip-sample junction at small separations of the probe apex. The likely contribution of local electron repulsion to the image is best interpreted in terms of EEDs, rather than from an analysis of the TED. Mapping of the short-range force and potential energy landscape shows that the tip-sample response at the positions of the intermolecular H bonds and intramolecular C–C bonds is surprisingly similar in terms of maximum attractive force, binding energy and force gradient. That both intramolecular and intermolecular contrast can be achieved on a silicon, rather than a metal, surface—and with a very different type of tip functionalization strategy than the protocols used for ultrahigh resolution DFM imaging to date^13^,^14^,^19^,^26^—demonstrates the broad applicability of the imaging mechanism pioneered by Gross *et al*. The results described here lead us to expect that imaging of inter and intramolecular features in parallel will be attainable for a broad range of molecule-substrate systems, although the detailed interpretation of those images will necessitate explicit consideration of the repulsive forces at the tip-sample junction and the relaxation of the apex of the functionalized probe.

## Methods

### Instrumentation and sample preparation

A commercial (Omicron Nanotechnology) ultrahigh vacuum and low temperature dynamic force microscope was used for imaging and spectroscopy at 5 K and 77 K. qPlus sensors with tungsten tips (also from Omicron Nanotechnology) were prepared for atomic and (sub)molecular resolution imaging by the application of voltage pulses and controlled crashing into the Ag:Si(111)-(√3 × √3)R30° surface. The Ag-√3 surface was generated by exposing a Si(111)-(7 × 7) sample, held at a temperature of ~550 °C, to a flux of Ag from a Knudsen cell. NTCDI molecules were also deposited from a Knudsen cell (temperature ~230 °C) onto the Ag-√3 surface, which was at room temperature.

### Atom-tracking-enabled force spectroscopy

Thermal drift was compensated at both 5 and 77 K using an atom tracking system developed at the University of Mainz^23^, interfaced with the Omicron MATRIX electronics. This system both measured the relative tip-sample drift/creep and applied feed-forward corrections. Full details of the force field acquisition, and the protocols used to extract the short-range forces from the frequency shift data, are given in the Supplementary Methods.

### Density functional theory calculations

DFT calculations were carried out using the CP2K code^27^,^28^. The algorithm implemented in the code is based primarily on using Gaussian basis set for evaluating most terms in the Hamiltonian; however, an on-the-fly conversion is made to a plane wave basis set to calculate the electrostatic energy. Goedecker, Teter and Hutter pseudopotentials and the Perdew–Burke–Ernzerhof generalized gradient approximation method^29^ were used. In all calculations the dispersion interaction was approximately taken into account using the DFT-D3 method as suggested by the study by Grimme *et al.*^30^ Geometry relaxation in most calculations was run until the forces on atoms that were allowed to relax were no more than 0.01 eV/A. Full details of the tip and sample geometries are given in the Supplementary Information file. Electronic density plots were generated using the XCrySDen code^31^.

## Author contributions

A.M.S., S.P.J. and P.M. conceived and designed the experiments. A.M.S. and I.L. carried out the experiments. S.P.J., H.S. and L.K. designed the DFT calculations. S.P.J., L.K., H.S., Y.W. and J.W. performed the DFT calculations. A.M.S. analyzed the experimental data. S.P.J., H.S. and L.K. analyzed the results of the DFT calculations. A.M.S., S.P.J., and P.M. wrote the paper. N.R.C. provided materials. P.R. contributed analysis and data acquisition tools.

## Additional information

**How to cite this article:** Sweetman, A. M. *et al.* Mapping the force field of a hydrogen-bonded assembly. *Nat. Commun.* 5:3931 doi: 10.1038/ncomms4931 (2014).

## Supplementary Material

Supplementary Figures, Methods, Discussion and References Supplementary Figures 1-17, Supplementary Methods, Supplementary Discussion and Supplementary References

Supplementary Movie 1 Mapping the 3D force-field of a hydrogen-bonded assembly #1

Supplementary Movie 2 Mapping the 3D force-field of a hydrogen-bonded assembly #2

Supplementary Movie 3 Series of constant height frequency shift images acquired at progressively small tip-sample separation.

Supplementary Movie 4 Series of constant height frequency shift images acquired above two NTCDI molecules pinned at a defect.
