# Supplementary material for: Mapping the force field of a hydrogen-bonded assembly
Source: Nat Commun. 2014 May 30;5:3931. doi: 10.1038/ncomms4931 (PMC4050271; doi:10.1038/ncomms4931)
Supplement: Supplementary Figures, Methods, Discussion and References — Supplementary Figures 1-17, Supplementary Methods, Supplementary Discussion and Supplementary References [file ncomms4931-s1.pdf]

SUPPLEMENTARY FIGURES

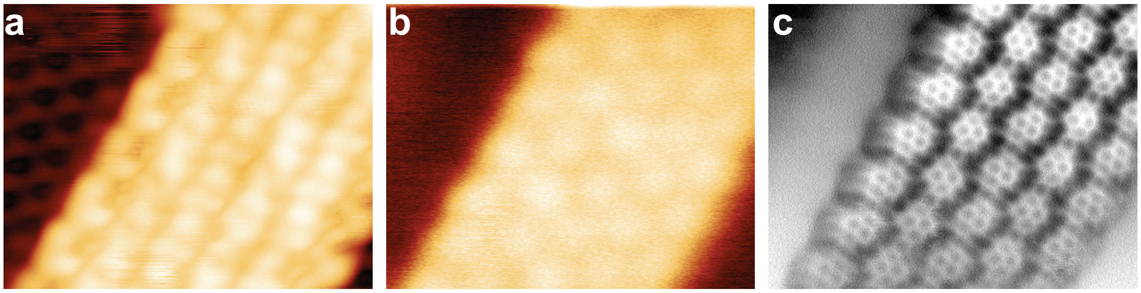

Supplementary Figure 1. **a)** Constant current feedback STM image of an NTCDI island. V_{gap} = +2V, I_t = 20 pA, oscillation amplitude (A_0) = 0.275 nm, **b)** Constant Δf feedback DFM, Δf = -13Hz, A_0 = 0.275 nm, **c)** Constant height DFM at Δz = -0.1nm, A_0 = 0.275 nm. Image size 7.2 x 5.5nm. Data collected at 77K.

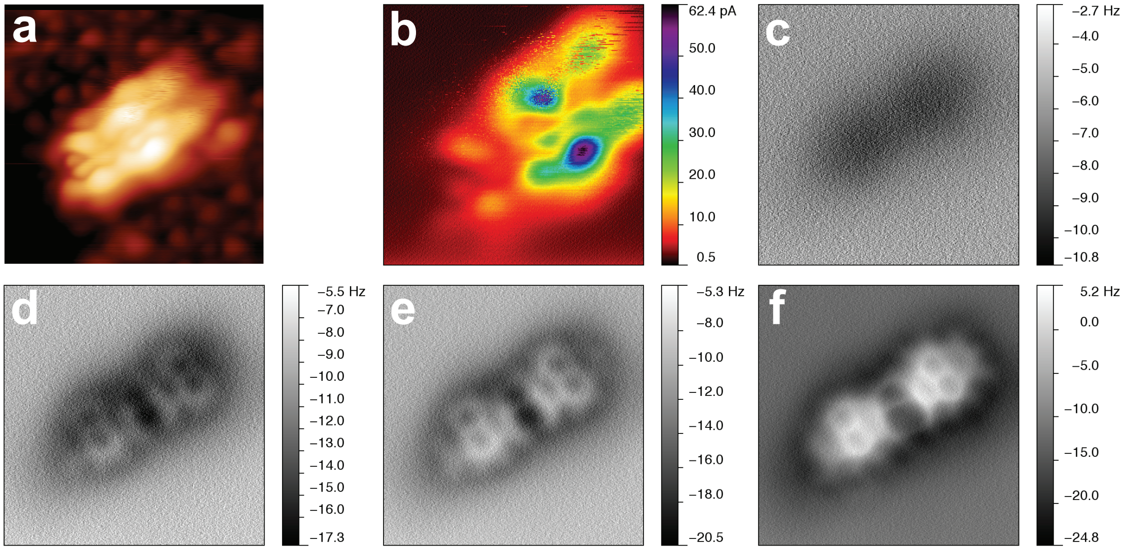

Supplementary Figure 2. **a)** Constant current feedback STM, V_{gap} = +1.5V, I_t = 20 pA. **b)** constant height tunnel current image, V_{gap} = +1V, A_0 = 0.275 nm. **c)** Constant height DFM at Δz = +0.055nm, A_0 = 0.11 nm. **d)** Δz = -0.077 nm. **e)** Δz = -0.11 nm. **f)** Δz = -0.175 nm. **b)**-**f)** Image size 2.6 x 2.6nm. Data collected at 5K.

A

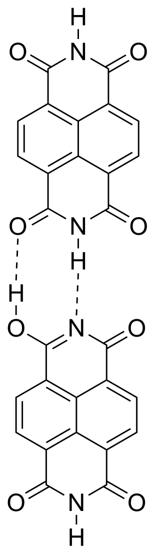

Supplementary Figure 3. **a)** Model showing possible hydrogen bonding configuration between an imide (top) and a tautomer (imidic acid - bottom).

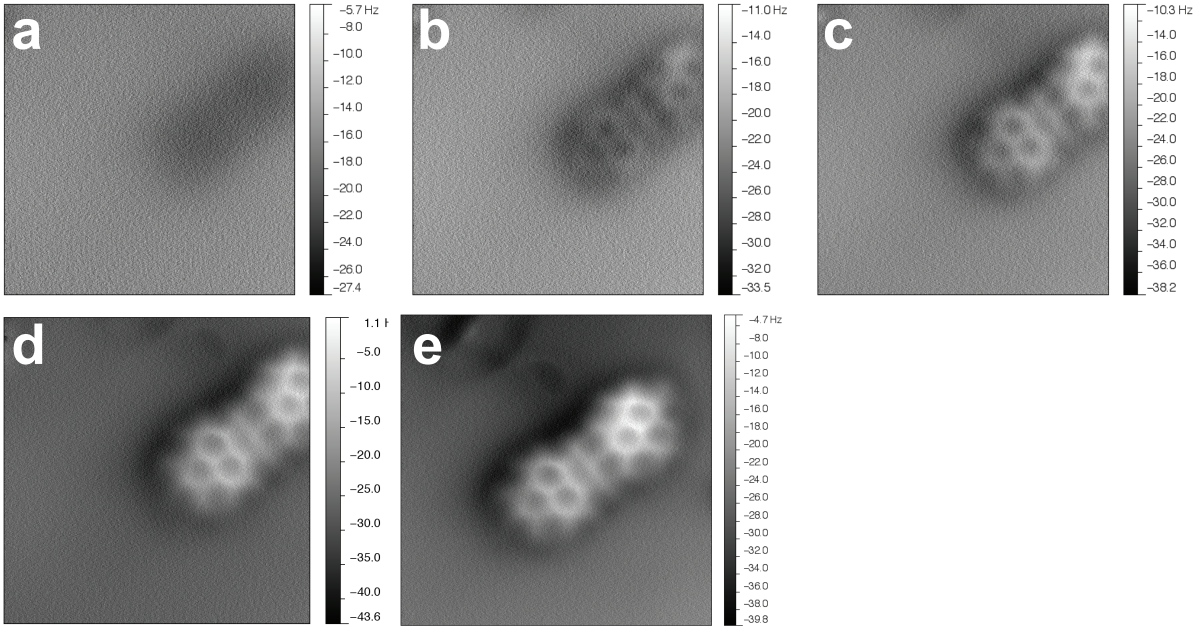

Supplementary Figure 4. Pair of isolated molecules imaged in constant height DFM. $A_0 = 0.11$ nm **a)** $\Delta z = 0$ nm. **b)** $\Delta z =$ -0.05nm. **c)** $\Delta z =$ -0.1 nm. **d)** $\Delta z =$ -0.15 nm. **e)** $\Delta z =$ -0.15 nm. Image size 2.8 x 2.8nm. Data collected at 5K.

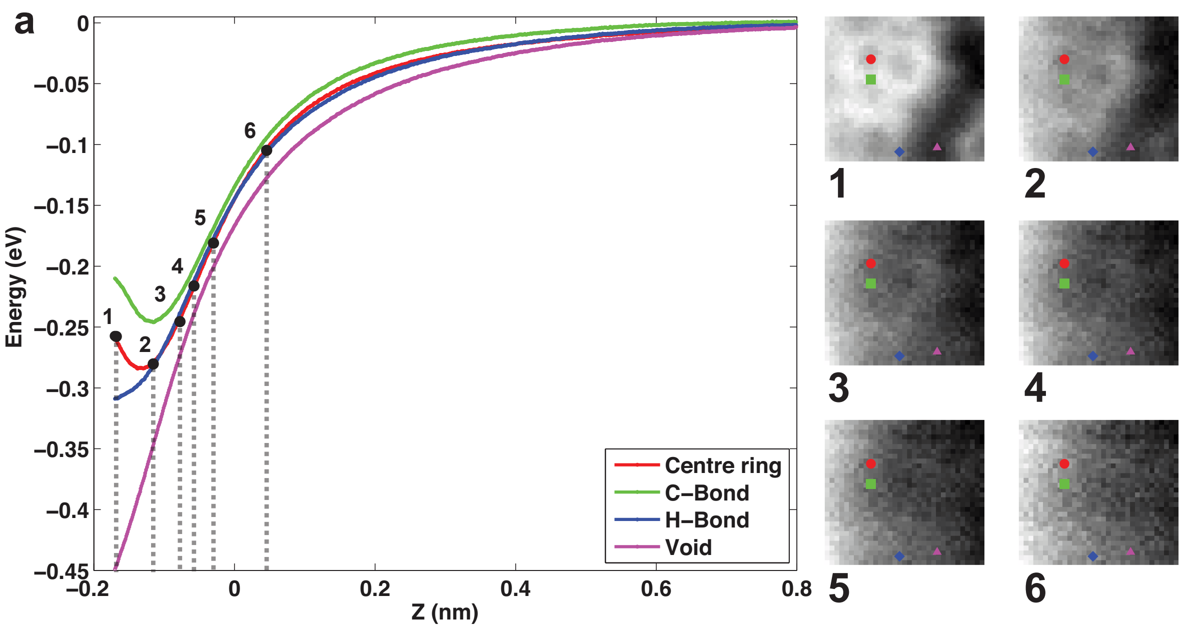

Supplementary Figure 5. Left: *U(z)* curves taken at selected points from the 3-D potential map (position shown in x-y potential maps on right). Right: x-y potential maps taken from the full dataset. The height of each x-y potential map is indicated by the position of the dotted black lines on the force graph. Image parameters: 33 x 30 pixel image. Image size: 1 nm x 0.9 nm Full $\Delta f(z)$ range -0.17 nm to +2 nm, 2000 points, 20 ms integration time per point. Temperature 5K. $A_0 = 0.11$nm

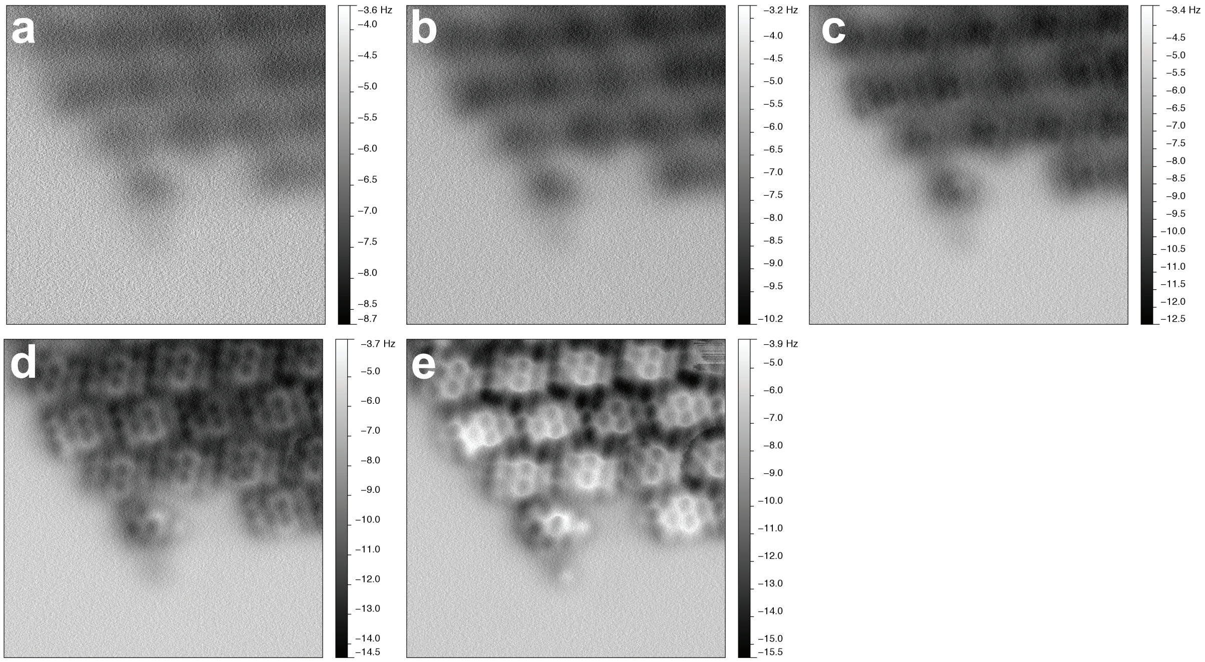

Supplementary Figure 6. Constant height slices showing overview of NTCDI island acquired before 3-D spectroscopy. **a)** Δz = +0.01nm, **b)** Δz = -0.03nm, **c)** Δz = -0.07nm, **d)** Δz = -0.11nm, **e)** Δz = -0.15nm, A_0 = 0.11 nm. Image size 5.1 x 5.1nm. Data collected at 5K.

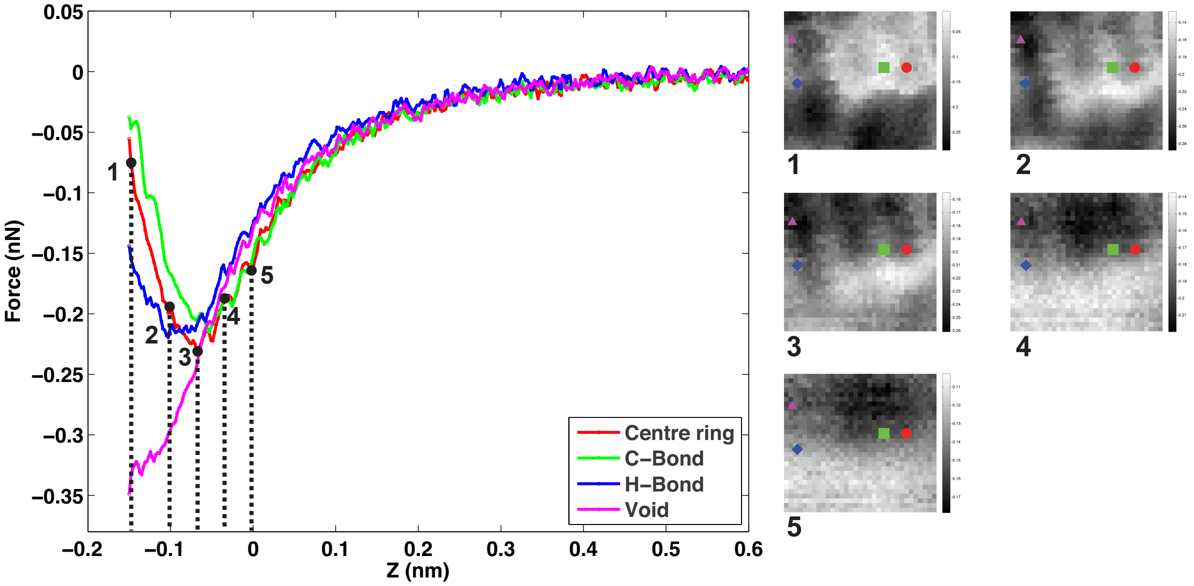

Supplementary Figure 7. Left: *F(z)* curves taken at selected points from the 3-D force map (positions are shown in x-y force maps on right). Right: x-y force maps taken from the full dataset. The height of each x-y force map is indicated by the position of the dotted black lines on the force graph. Image parameters: 41 x 41 pixel image. Image size: 1 nm x 1nm. Full $\Delta f(z)$ range -0.15 nm to +2 nm, 1000 points, 20 ms integration time per point. Data collected at 5K.

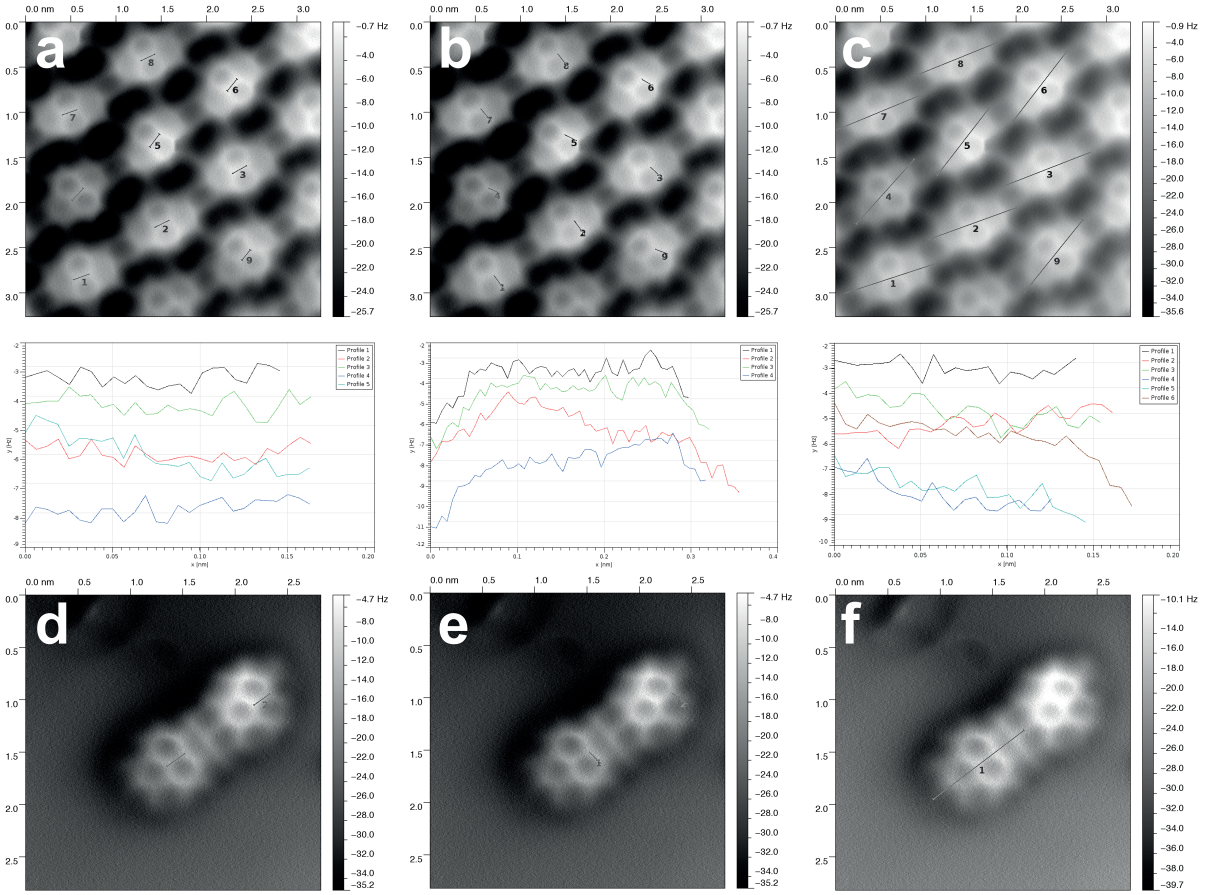

Supplementary Figure 8. Bond length measurements on NTCDI molecules. Each figure shows line profiles in the estimated positions of the intermolecular features. **a)-c)** within and NTCDI island, **d)-f)** Isolated molecules. **a)** Central C=C bond. Average bond length = 0.17(5)nm. DFT calculated bond length = 0.144nm. **b)** ‘Shoulder’ C=C bond. Average bond length = 0.15(1)nm. DFT calculated bond length = 0.144nm. **c)** NTCDI length. Average length = 1.0(2)nm. DFT calculated length = 0.91nm. **d)** Central C=C bond. Average bond length = 0.19(5)m. **e)** ‘Shoulder’ C=C bond. Average bond length = 0.15(6)nm. **f)** NTCDI length. Length = 1.(1)nm. Image size shown on axis. Data collected at 5K.

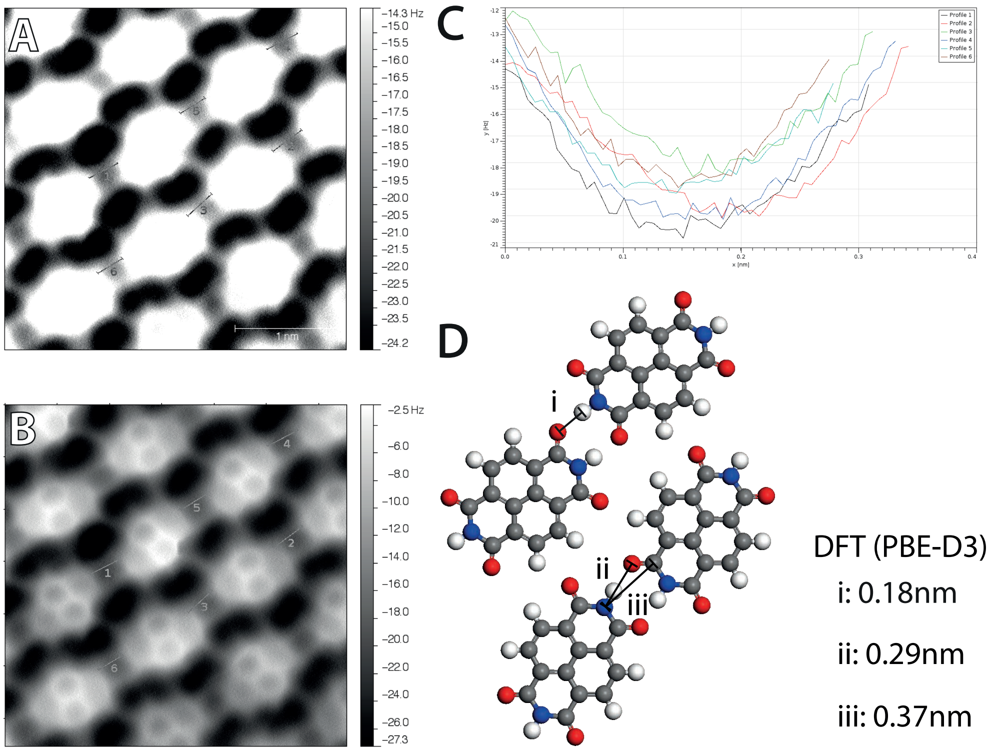

Supplementary Figure 9. Length measurement on hydrogen bond features. (A) Contrast enhanced image of the hydrogen bonded network. (B) The same image as A shown with normal contrast. (C) Line profiles shown in the estimated positions of the intermolecular features. Average length = 310(40)pm. (D) Schematic showing the calculated hydrogen bond length in (i) as 180pm. Atomic separations (ii) and (iii) are marked as a comparison for the experimental measurement.

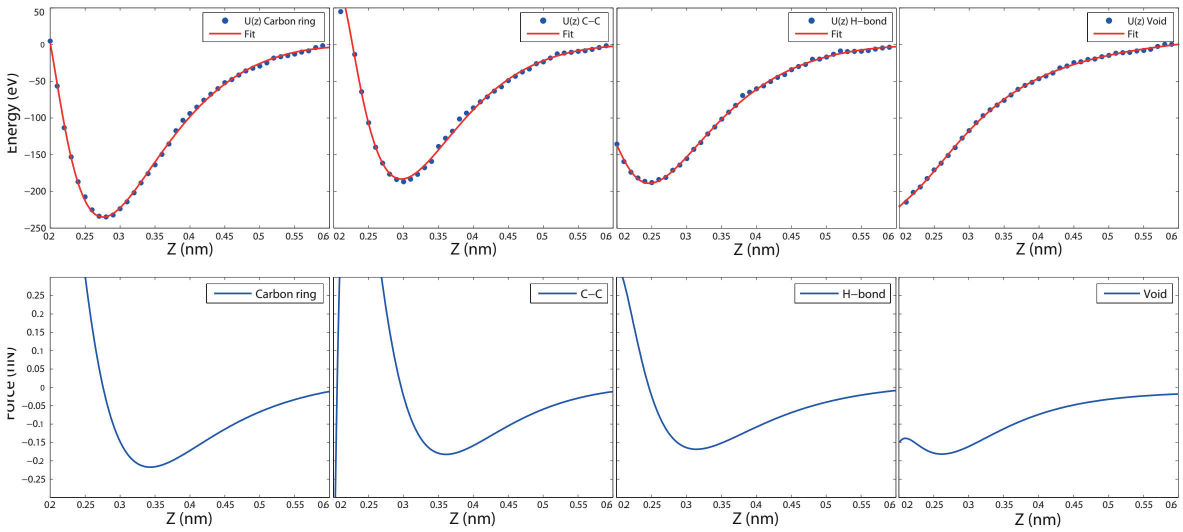

Supplementary Figure 10. Calculation of *F(z)* from simulated energy, *U(z)*. *U(z)* was fitted with a 5th order inverse power law and differentiated to obtain *F(z)*. Panels show example *U(z)* fits and corresponding *F(z)* for the O-down tip shown in Figure 3a of the main paper.

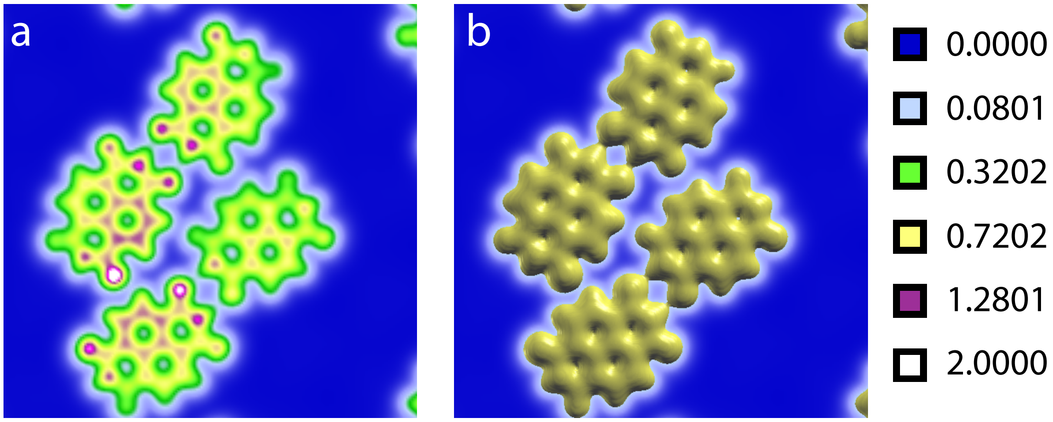

Supplementary Figure 11. Total electron density slices taken in x-y plane positioned 500pm above the NTCDI molecular plane. **a**, Total electron density plotted to highlight the differences in charge in the H-bonding regions and carbon backbone. **b**, The same plot as **a** with the addition of a 3D isosurface plotted at 0.08 e/a_0^3, the minimum at which we observe charge in the H-bonding regions (see Legend on the right).

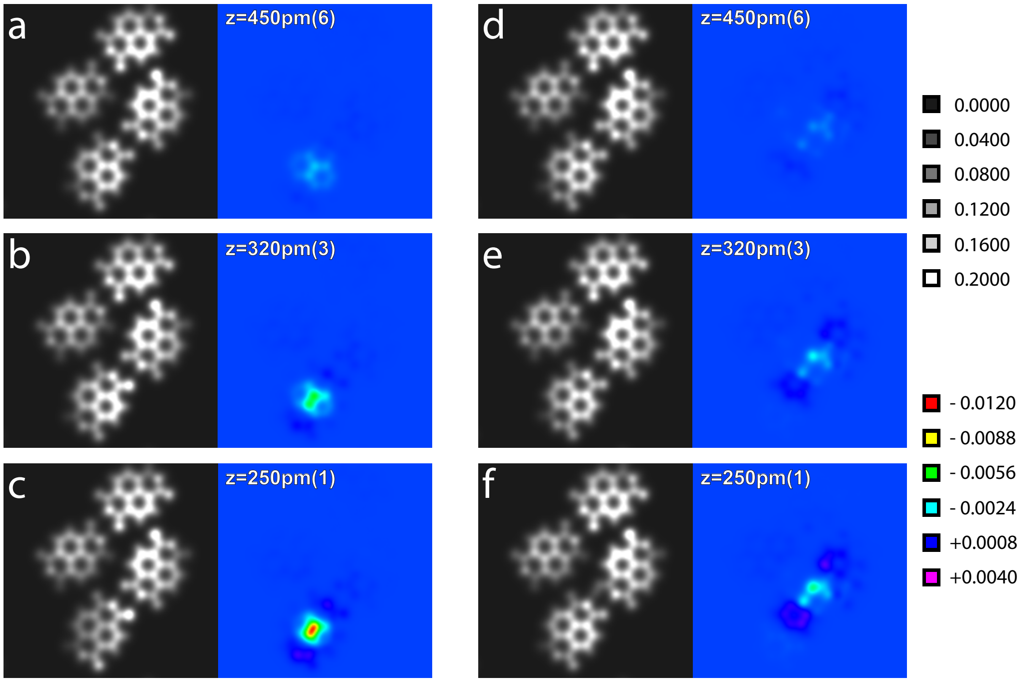

Supplementary Figure 12. DFT calculated total electronic density and electron density difference plotted in x-y plane 100pm above the molecular plane for tip z positions corresponding to Figure 4 of the main paper. TED (left) and EDD (right) plotted during approach of an O-down NTCDI tip at the C-C location on an NTCDI molecule at tip-sample separations of **a)**, z=450pm, **b)**, z=320pm and **c)**, z=250pm. **d)-f)**, Equivalent plots for the H-bond location.

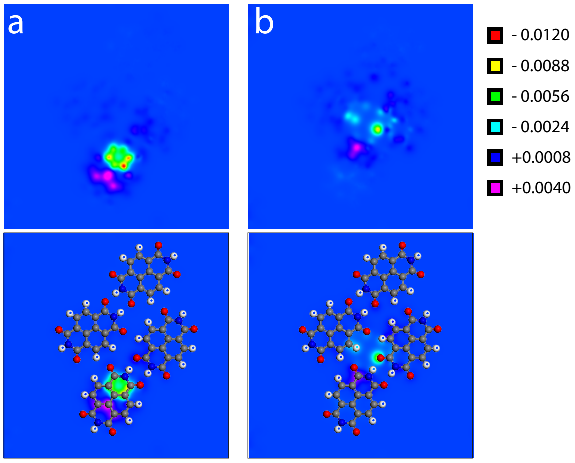

Supplementary Figure 13. Electron density difference calculated for **a)**, the Carbon Ring and **b)**, Void positions for an O-down NTCDI tip at a tip-sample separation of z=250pm (plotted at 50pm above the molecular plane, the same as Figure 4 of the main text). In the bottom images the molecular models are superimposed on the top images to show the position of the calculated contrast within the molecular island.

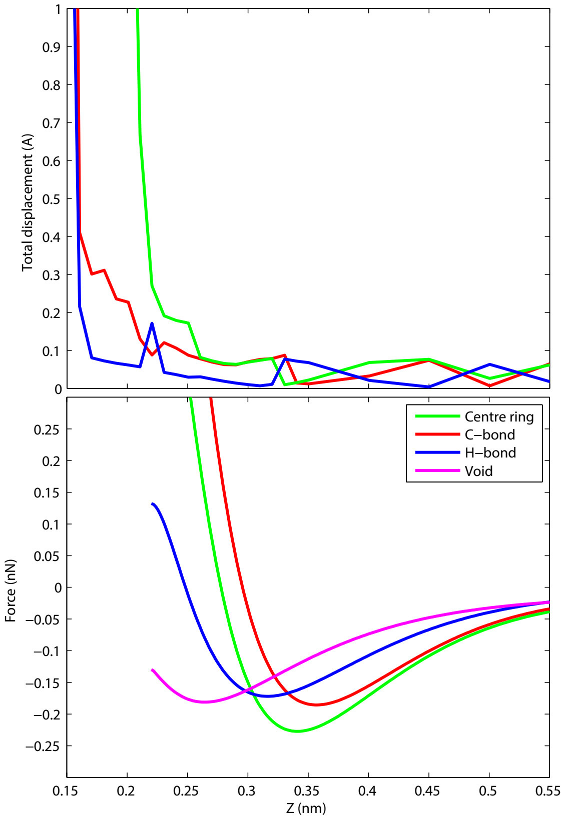

Supplementary Figure 14. Calculated AFM force and atomic displacement of the NTCDI oxygen-down tip during simulated spectra. The atomic displacement is calculated by taking a root of the sum of squares of the x, y and z displacements.

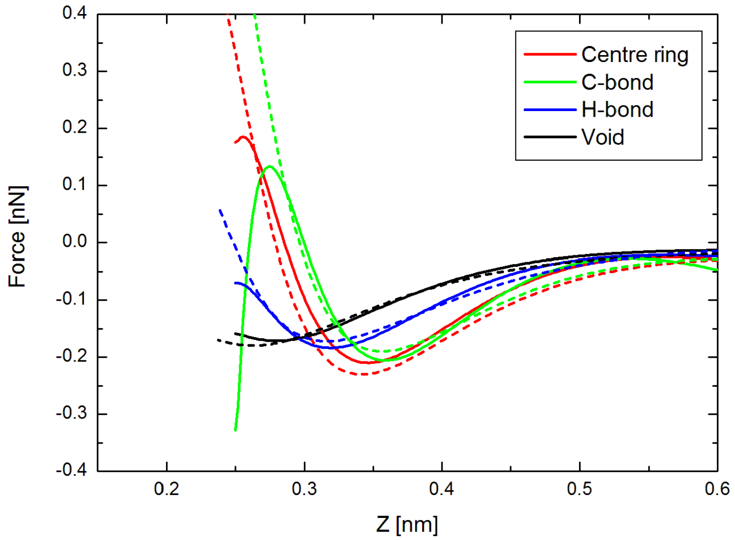

Supplementary Figure 15. Calculated F(z) for CO terminated tip overlaid with O:NTCDI from Figure 3A of the main text. Oxygen-down NTCDI data is shown as dashed lines with CO data shown as solid lines.

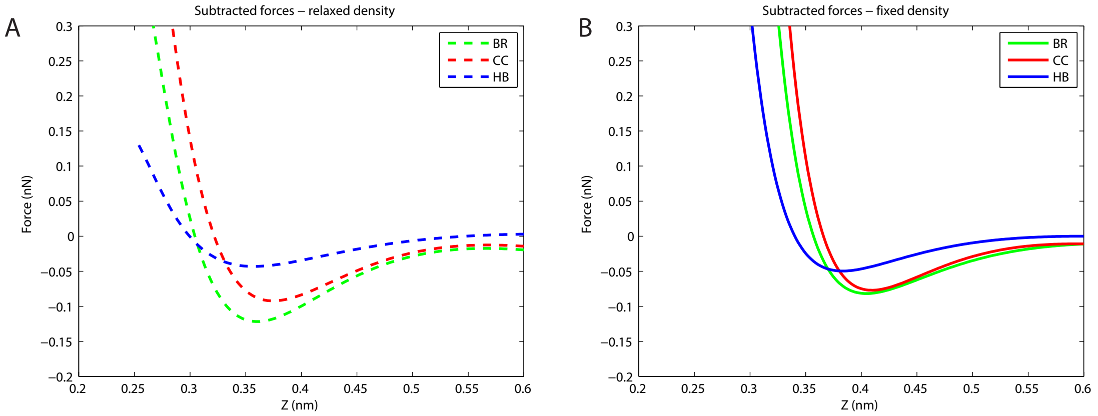

Supplementary Figure 16. Calculated F(z) for an oxygen down NTCDI tip (A) with normal atomic and electronic relaxation and (B) the constrained system with no allowed relaxation of the density or atomic positions.

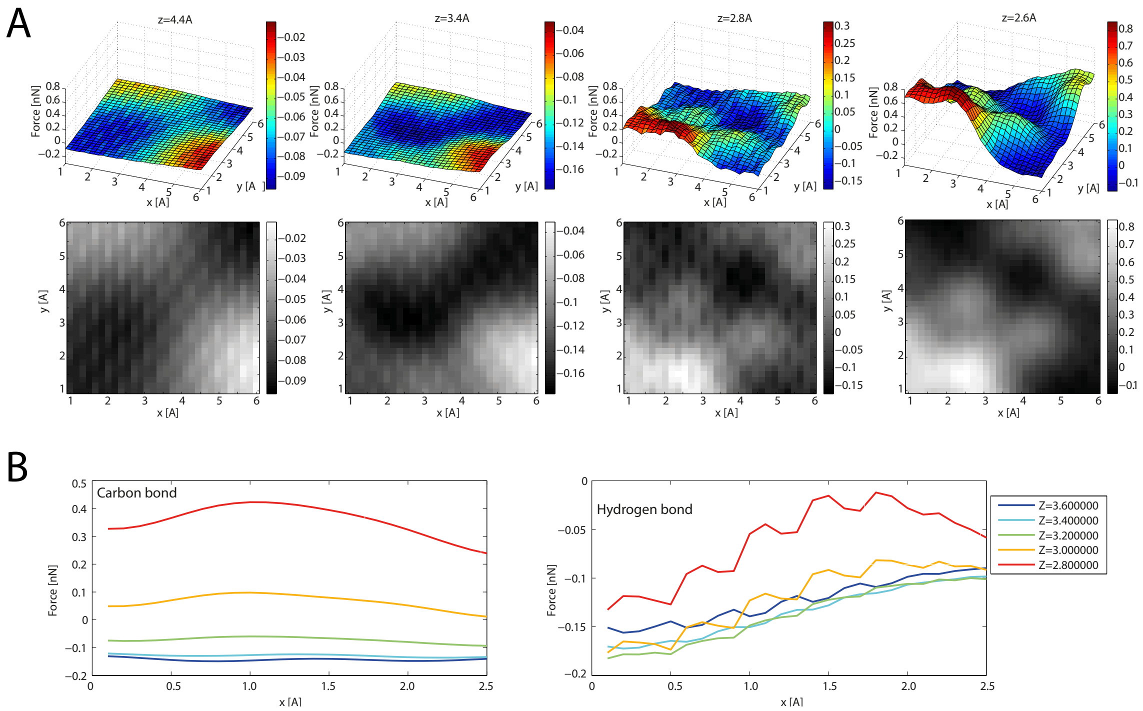

Supplementary Figure 17. (A) Extracted constant height slices taken from simulated 3D force data at tip-sample separations of z=4.4Å, 3.4Å, 2.8Å and 2.6Å. Data is shown as both 2D intensity and 3D contour maps. (B) Force line profiles calculated at decreasing tip-sample separation above the C-bond and H-bond position as shown in Figure 4E of the main text.

SUPPLEMENTARY METHODS

Experimental methods

All experiments were carried out in ultrahigh vacuum (UHV) using an Omicron Nanotechnology GmbH LT STM/AFM in the qPlus configuration, using MATRIX electronics as the control system. Clean Si(111) 7 x 7 surfaces were prepared by the standard method of degassing and then flash annealing (boron doped: 0.020 Ohm cm) silicon wafers in UHV. The Ag:Si(111)-($\sqrt{3} \times \sqrt{3}$)R30 surface was then prepared by depositing silver from a Knudsen cell onto the silicon surface heated to approximately 550°C. After successful silver termination, NTCDI molecules were deposited from a boron nitride crucible onto the substrate from a different Knudsen cell (heated to approximately 230°C), with the Ag:Si(111) silicon sample at room temperature. After deposition the sample was transferred immediately into the low temperature cryostat for imaging.

Experiments were performed at either $\sim$ 5 K (using liquid helium) or $\sim$ 78 K (when using liquid nitrogen). Although operating at 5 K offered significant advantages in terms of reducing drift and creep as well as improving the overall noise level, we did not otherwise observe any qualitative differences in the measurements performed at different temperatures.

We used commercial qPlus sensors supplied by Omicron Nanotechnology, with an electrochemically etched tungsten wire attached to one tine of the tuning fork as a tip. These were introduced into the scan head without any further conditioning. The tips were then prepared on clean silicon surfaces by standard STM tip preparation (tip pulses and controlled crashes into the surface) until good STM and DFM resolution was obtained. Consequently, before we start imaging the NTCDI molecules, we expect that our tips are silicon, rather than tungsten, terminated.

For the sensors used in this study, we recorded resonant frequencies of the order of 25 kHz, and on the basis of previous stiffness measurements of similar sensors [1] assumed an effective stiffness of approximately 2000 N/m during force inversion calculation. The oscillation amplitude of the sensor was calibrated using the Giessibl normalised frequency shift method. We expect both of these values are subject to a (systematic) uncertainty of approximately 10%.

Experimental techniques

When imaging the islands of NTCDI, typically the region of interest was first imaged in STM, and left to scan in order to minimise drift and creep, before subsequently attempting to image the same region in constant height DFM. When imaging in constant height DFM, we ensured the gap voltage was set to 0V (i.e. no detectable tunnel current) in order to eliminate any effect of either electronic crosstalk [2], or the so-called phantom force [3]. For STM measurements the sample was biased and the tip held at ground.

When imaging the molecular islands on the silver-terminated surface in STM we observed a large number of spontaneous changes in resolution, suggesting that the tip was picking up or depositing material from the substrate. It is partly on this basis that we suggest that our tips become NTCDI-terminated during the initial STM imaging.

Atom tracking and 3-D forcefield measurement

At both 5 K and 77 K we used a home-made atom tracking system developed by Rahe et al. at. [4], interfaced with the Omicron MATRIX electronics, to both measure the relative tip-sample drift/creep and subsequently apply feedforward correction. At liquid helium temperatures this was not necessary in the case of taking a single constant height image, but was required over the long periods of time needed to acquire the 3-D datasets presented (see below for more details). At liquid nitrogen temperatures we found the use of feedforward correction was essential in order to allow reliable constant height imaging of the molecules and surface, even in the case of single scans.

In order to acquire the 3-D force fields we used a grid spectroscopy protocol combined with the atom tracking previously described by Rahe et al. [4]. In brief, once the tracking parameters had been determined, the tip was left for some time tracking a single molecule. To perform a measurement the tracking and dither would be disengaged and the tip would move to a predefined position and perform a single $\Delta f(z)$ spectroscopy measurement, the tip would then move back to the original location, reengage the dither, and re-enter tracking. We note additional delays were incorporated during repositioning steps to reduce the effect of piezo creep. This procedure was repeated for every pixel of the 3-D grid. The x-y position of the tip was recorded each time the tip re-entered tracking, and the feed-forward vector updated every 10-30 minutes based on the recorded motion in that time. A typical 3-D

grid would entail between 1000 and 2000 $\Delta f(z)$ measurements depending on the size and pixel density. We prefer to construct the 3-D force fields using this protocol rather than the alternative x-y "slice" methods as we believe that there is less potential for introducing artefacts either from drift correction, or errors in the amplitude/phase signals due to the high Δf corrugation present when imaging in constant height. During $\Delta f(z)$ measurements integration times and PLL/AGC settings were selected such that the phase and amplitude signals of the PLL remained constant within the instrumental noise. The use of the atom tracking to correct for long-term drift, and to continuously relocate the tip over the molecule, was necessary due to the very long acquisition times required in order to record these datasets (approximately 29 hours per 3D grid). For constant height images taken using atom tracking all Δz values quoted are relative to the height of the tip in Δf feedback during tracking.

F(z) curves were extracted from the $\Delta f(z)$ spectroscopy by subtracting $\Delta f(z)$ measurements taken off the island from $\Delta f(z)$ measurements on top of the molecules. This "on minus off" method (used for example by both Lantz et al, and Ternes et al [5, 6]) produced a "short-range" Δf that was then inverted to force by the Sader-Jarvis method [7]. Although all measurements were acquired in constant height we note it was necessary to process the 3D data to remove variation in Δf due to drift in the frequency of the PLL (of order 40-100mHz over a 30 hour period). After this processing step we ensured the long range part (i.e. those parts of the curve not showing site specific contrast, usually over +1 to +1.5nm from the feedback position) of the $\Delta f(z)$ curves were aligned using a least mean squares fitting method in order to check the drift in Δf had not significantly altered the feedback position during atom tracking. The alignment checked by this method was generally better than 5pm, below our vertical noise limit in Δf feedback. Consequently, the grid datasets could be processed as constant height datasets within our experimental noise.

As a result of the "on minus off" method the calculated force values only include site-specific interactions, as long-range van der Waals and electrostatic interactions are subtracted out. We explicitly note, however, that any site specific van der Waals interactions (that is to say, the short range dispersion attraction between the apex of the tip and the molecule on the surface) are still included (and, as described below, are also accounted for in our density functional theory calculations). The "on" and "off" measurements are both taken over the same range of z, but only the "on" measurement contains contributions from the short range (site specific) force. Following subtraction of the "off" curve, we therefore obtain the short range signal, which is directly comparable to single DFT *F(z)* calculations.

Simulation methods

Calculations were performed using an *ab initio* density functional theory (DFT) method and the CP2K code [8, 9]. The algorithm implemented in the code is based primarily on using a Gaussian basis set for evaluating most terms in the Hamiltonian. However, an on-the-fly conversion is made to a plane wave basis set to calculate the electrostatic energy. Goedecker, Teter, and Hutter (GTH) pseudopotentials and the PerdewBurkeErnzerhof (PBE) generalized gradient approximation method [10] were used. In all calculations dispersion interactions were approximately taken into account using the DFT-D3 method due to Grimme et. al.[11]. Geometry relaxation in most calculations was run until the forces on atoms that were allowed to relax were no more than 0.01 eV/A.

Several tips were used based on two models: a Si pyramidal tip terminated with either (i) H or (ii) Ag atoms, and a vertically oriented NTCDI molecule facing the surface in two ways: (iii) with its N-H group facing towards the surface; (iv) with one of its O atoms facing towards the surface, the H- and O-down terminations are shown in Figure 3b,d of the main text. In calculations with the Si pyramidal tip the top two layers of atoms were fixed and the bottom atoms were allowed to relax. In calculations with the NTCDI tip, various numbers of atoms in the top part of the molecule were fixed. However, at least sixteen atoms in the bottom half of the molecule were free to relax in all our calculations.

Two atomic layers were used when modeling the Ag-terminated Si surface as shown in Figure 3b of the main text. The bottom Si layer was fixed and terminated with H atoms and the top terminated with Ag to model the Ag:Si(111)-($\sqrt{3} \times \sqrt{3}$)R30° IET reconstruction. Due to the lack of commensurability between the NTCDI network and the underlying substrate we were unable to model a periodic NTCDI assembly. As we are limited to using relatively small unit cells due to computational expense, placing a periodic network of NTCDI molecules on the surface would create unphysical compression or stretching of the molecules in the monolayer. To avoid this problem, an isolated cluster of four NTCDI molecules was considered using a rather large unit cell to avoid spurious interaction between neighboring images of the clusters, see Figure 3c of the main paper. This cluster corresponds to a fragment of an actual island consisting of two rows of flat molecules with two molecules in each row. The molecules in each row are arranged head-to-tail and bound by a double N-H$\cdots$O hydrogen bond. The interaction between molecules in different rows corresponds to a combination of weak C-H$\cdots$O hydrogen bonds and dispersion (van der Waals) forces.

The molecules in the cluster were initially fully relaxed. Then, prior to spectroscopy calculations, we fixed a number of atoms at the periphery of the cluster to emulate their relative immobility due to interaction with the rest of the island, not explicitly modelled. The spectroscopy calculations were performed in the following way. Initially we positioned the simulated tip at an initial height of +0.6nm with respect to the surface molecule at several sites marked in Figure 3c of the main paper. The tip-sample separation was then reduced in small quasi-static steps of 0.01nm towards the surface, relaxing the system geometry at each point. The calculated total energies were then fitted with a sum of inverse power law contributions, up to fifth order (of form $a+b/x+c/x^2+d/x^3+e/x^4+f/x^5$) and differentiated to obtain the tip-sample force as shown in Supplementary Figure 10. Other functions, such as the Lennard-Jones and Morse potentials, were found to give poorer fits due, in part, to their lack of any consideration of atomic relaxation in the repulsive part of the potential. Density difference plots were obtained by subtracting from the electron density of the tip-surface system the electron densities of the isolated tip and of the surface in the geometry of the combined system.

SUPPLEMENTARY DISCUSSION

Additional experimental results

STM to DFM to constant height DFM

Supplementary Figure 1 shows an example of a transition from STM to constant Δf feedback DFM in the same region. Although sub-molecular resolution is obtained on the NTCDI island in STM, the orientation of the molecules, and the origin of the contrast are not immediately clear. In constant Δf DFM molecular resolution is obtained, but the molecules appear as diffuse blobs, with no internal contrast. In constant height mode, at a tip sample distance sensitive to the repulsive interaction, the internal structure of the molecules, their orientation, and the features at the expected positions of intermolecular hydrogen-bonds are clearly resolved. We note that in this example a clear asymmetry in the tip is obvious due to the difference in contrast on the opposite edges of the island.

Imaging low coverages of NTCDI on silver-terminated silicon

As previously shown, at larger coverages NTCDI readily forms large islands on the silver terminated silicon surface. At low coverages the molecules are mostly found absorbed either individually or in small clusters at defects on the surface. These pinning sites may include unpassivated silicon atoms or disordered silicon - silver clusters, and, consequently, the exact nature of the binding of the molecules to the substrate is not well defined in these instances. However, it is almost certainly much stronger than the weak van der Waals forces underpinning the molecule-substrate interaction on the clean, passivated Ag:Si(111)-($\sqrt{3} \times \sqrt{3}$)R30 regions.

Due to their pinning at the defect sites we sometimes find molecules adsorbed in less energetically favourable configurations than the hydrogen bonded configuration found in the islands. Supplementary Figure 2 shows a sequence of images showing imaging of a pair of molecules after a low coverage deposition of NTCDI. The constant current STM and constant height STM show contrast similar to that observed within the islands, although the increased bonding to the substrate (due to the pinning at the reactive defect sites) likely perturbs the electronic structure of the molecules more strongly than those absorbed on the silver. The constant height DFM imaging of the same molecules reveals the same carbon backbone structure as observed in the images of the large islands. The pinning of the molecules in this case means that the molecules are arranged in an unfavourable "end-on" configuration. In this instance we also observe *intramolecular* contrast between the molecules. Here there are two possible explanations of the contrast. The first is that we are observing bonding between the NTCDI, and one of its tautomers (see Supplementary Figure 3). The other is that both molecules have the same tautomerisation and that the close spacing of the molecules in this instance produces contrast between the molecules similar to that observed in the hydrogen bonding configuration, although we note explicitly that in the configuration shown no hydrogen bonding would occur between the aligned groups. Consequently, we suggest a degree of caution must be applied to the interpretation of inter-molecular contrast as it is not trivial to see how repulsive effects purely due to the close proximity of the molecules could be distinguished from hydrogen bonding effects.

Supplementary Figure 4 shows another two molecules obtained at low coverage, in a similar bonding configuration to Supplementary Figure 2, the same sub-molecular resolution is obtained. The right-hand molecule appears slightly distorted at the far end, possibly due

to its bonding to the substrate.

Mapping the potential field

In addition to the force-field presented in the main paper we also calculated the potential energy of interaction between the tip and molecule (Supplementary Figure 5). Qualitatively we observe the same contrast in the x-y maps as for the force, and we observe reasonable quantitative agreement between the experimental potentials and the energies calculated in our DFT simulations. However, on a cautionary note, we find that both the magnitude, and shape, of the potential curves are much more sensitive to any variation in the alignment of the curves, or variations in experimental noise, than the corresponding force curves. This can be readily understood from the relationship between force and potential, $F(z) = -dU/dz$, and thus although the force measurements are therefore 'noisier' "point by point", they are much less sensitive to any 'integrated' variation in the tail of the potential. Conversely, it is extremely instructive to examine the potential curves during data processing, even if the quantity of interest is the force, as any variation is often much clearer in the potential than the force.

Additional 3-D dataset on NTCDI island on silver-terminated silicon

In this section we show an additional dataset acquired using the same method as for the 3-D dataset in the main paper, in order to show the reproducibility, both qualitatively and quantitatively, of the contrast and the measured tip-molecule forces. Supplementary Figure 6 shows constant height overview scans of the NTCDI island acquired before the grid data presented in Supplementary Figure 7.

Supplementary Figure 7 shows individual force spectra at selected points and a series of x-y force slices from a 3-D force spectroscopy dataset acquired on the island shown in Supplementary Figure 6. Although this set does not enter as far into the repulsive regime as the dataset in the main paper, the same contrast, and the same ordering of the force curves in the repulsive regime is apparent. Additionally the magnitude, and differences between the individual force curves, agree quantitatively with the dataset from the main paper, suggesting a very similar tip termination must be responsible.

Deliberate tip termination with NTCDI

Due to the high coverages of NTCDI required to form the hydrogen bonded assemblies, deliberate tip functionalisation via pick up of an individual molecule proved challenging. Unlike experiments conducted with CO there is no clear indication that a molecule has been successfully transferred onto the STM/AFM tip (e.g. inversion of contrast in STM). Similarly, STM images of NTCDI islands are complicated to interpret and cannot be used to determine whether the tip has become sharper through pickup of the molecule - as is the case for other tip terminations such as Br and Xe [12]. Consequently, the primary method used to check for tips capable of submolecular resolution was to directly attempt imaging in the constant height AFM mode. We therefore rely on corroborating information and comparisons with DFT simulations described elsewhere in the paper to determine our tip state.

Bond length measurements

In their recent work, Gross et al. [13] observed distortion of the measured carbon - carbon bond lengths during constant height imaging of planar molecules, due to the relaxation of the CO molecule adsorbed on the end of their tip. In their work the apparent carbon - carbon bond length were measured to be 45 – 90 % larger than their real size. In Supplementary Figure 8 we present measurements taken on NTCDI both in islands, and isolated pairs, and compare them to bond length distances calculated by DFT for a NTCDI molecule in the gas phase. Interestingly, we observe less distortion of the bond lengths than Gross et. al. for both cases, with experimental values which differ from the calculated values by as little as 5% in some cases. The largest deviation between the experimental and theoretical values we observe is 30%. This may suggest that the tip apex producing the contrast in our measurements is stiffer than the Cu - CO tip commonly used in these types of experiments.

Measurements of the hydrogen bond length are shown in Supplementary Figure 9. These, however, are much less straight forward to interpret. In our AFM images the periphery bonds of the NTCDI molecule, i.e. the C-H, N-H and C=O are not clearly observed. Determining the exact length of the observed intermolecular features is therefore prone to a degree of subjectivity. Exemplary line profiles depicting several measurements of the intermolecular features are shown in Supplementary Figure 9C marked at the positions shown in A and B,

which average a length of 310(40)pm. To facilitate comparison with the DFT simulations, calculated lengths for three separations are shown in Supplementary Figure 9D. Although the experimentally observed features appear larger than the calculated length of the hydrogen bond, they are likely overestimated due to the fact that no clear distinction can be made from the periphery bonds. Experimental line profiles have been included here for clarity, however, we stress again that accurate determination of the hydrogen-bond length from the images is problematic, and therefore should be treated with a degree of caution.

Atomic relaxations at very close approach

Due to the precision available at 5K temperatures, combined with the atom-tracking system, thermal drift was eliminated such that the variation in molecular resolution with scan height could be carefully examined. Particularly noticeable in Figure 2 of the main paper (see also movie file 3D grid Force 01) is a slight distortion of the NTCDI molecule when the tip height is reduced below -0.15nm, shown as point 1 in Figure 2. As all of our 3D data was collected using a grid spectroscopy protocol (see Section II.1), we can be confident that the distortions of the molecule are in fact real, likely due to the tip tilting under compressive strain at very close approach. It is important to note that we clearly observe *both* intramolecular *and* intermolecular features as early as the force turnaround suggesting that only a small amount of repulsion is required to distinguish these features.

To further investigate the effect of tip distortions we extracted the atomic displacements from our DFT calculated spectra. Shown in Supplementary Figure 14 is the calculated force spectra and atomic displacement for the NTCDI tip in the oxygen-down orientation at the primary locations investigated. The atomic displacement characterises the x-y-z movement of the lowest oxygen atom of the NTCDI-tip. Significant deformations of the NTCDI molecule are only observed when the tip-sample separation is <0.3nm, which corresponds to the same region of the experimental 3D force data where distortions are observed for the NTCDI molecule described above. As such our DFT calculations support the proposal that the distortions observed at very close approach for our data in Figure 2a of the main paper is a consequence of tip (and NTCDI island) relaxations. However, it is useful to note that the regime of very close approach corresponds to a significantly smaller scan height, around 50-100pm less, than what is required to observe the intramolecular and intermolecular resolution shown in this paper.

Movies

Movies of the 3D datasets presented in the main paper and supporting information are available as separate files to show the full evolution of the force contrast through the dataset.

Additional DFT results

Total electronic density calculations

To interpret the experimental images the total electron density (TED) was calculated for the simulated NTCDI island adsorbed onto the Ag:Si(111)-($\sqrt{3} \times \sqrt{3}$)R30° surface. Whilst reasonable agreement was found for the *intra*molecular resolution observed within each NTCDI molecule, the *inter*molecular resolution in the regions of H-bonding was not reproduced. The TED is plotted on a *linear* scale in Supplementary Figure 11a and b which shows that the density in the region of H-bonding is over an order of magnitude (∼0.08 versus ∼1.5 electrons/Bohr^3) smaller than that over the C-C bond regions. Supplementary Figure 11 highlights this disparity by plotting the minimum density isovalue at which charge is observed in the H-bond locations. As such, consideration of the TED is not sufficient to explain our *inter*molecular DFM observations. We note that in previous observations of intermolecular bonding [14] the calculated TED images required significant saturation of the scale bar for any intermolecular contrast to be visible, a process that is *not* required for the experimental DFM images.

Electron density difference

As discussed in the main paper, Supplementary Figure 12 shows calculated TED and electron density difference(EDD) plotted as an x-y slice positioned 100pm above the molecular plane. The electron density difference was obtained by first calculating the TED for the isolated surface (Ag- $\sqrt{3}$ + NTCDI molecular island) and also the isolated NTCDI tip at each step of the *F(z)* calculation. These two densities were then summed together and subtracted from the total density for the full relaxed system. Therefore the remaining subtracted electron density difference will describe the interaction caused by the presence of the tip, allowing us to examine its effect on the simulated system.

At this position the difference in electron depletion between the C-C and H-bond positions

appears to be greater than in the main text. This is because for the H-bond location the electron depletion is centred along the axis of the H-bond, in plane with the NTCDI, whereas for the C-C bond the depletion is stronger above the plane of the molecule (at 50pm as shown in Figure 4 of the main paper). It is interesting to note that in Supplementary Figure 12c the EDD above the C-C bond takes on a sharp appearance, similar to that observed in experimental images. We also note that in Supplementary Figure 12 f there appears to be enhancement of the TED in the H-bond location, however, this is a misleading observation. In Supplementary Figure 12 the x-y slices are plotted 100pm above the molecular plane, 50pm higher than that shown in Figure 4 of the main paper, whereas the DFM tip position (defined by the location of the lowest atomic core) is 250pm above the molecular plane. Therefore the position of the x-y slice at 100pm is high enough to intersect the electron density originating from the atoms within the DFM tip, which is unrelated to the tip-surface interaction. The enhancement is thus purely apparent and bears no relation to interpretation of our DFM images.

Supplementary Figure 13 shows the calculated EDD for (a) the carbon ring and (b) void positions marked in Figure 2 and Figure 3 of the main text, plotted with the same position and scale as that in Figure 4 and overlaid with the ball-and-stick model. Compared to the other positions the void location shows a dramatic reduction in the amount of electronic density redistribution from the presence of the AFM tip due to the absence of any NTCDI molecules or intermolecular bonding in this location. Furthermore, the observed density depletion occurs in locations other than below the AFM tip, and is in fact centred on the atomic cores of the surface NTCDI molecules. This supports our observation that the density observed for the H-bond location is in fact due to real interaction between the surface network hydrogen bonds **and** the AFM tip. For completeness, the Carbon Ring location is also shown. Here we observe that the density depletion is strongest over the individual carbon atoms, with an intensity between that of the C-C and H-bond locations, possibly reflecting the same ordering observed in the calculated *F(z)* curves at negative gradients.

Carbon monoxide tip calculations

We have shown that to achieve intra- and inter-molecular resolution in our experiments our tip is most likely terminated with a single NTCDI molecule oriented with its C=O bond pointing towards the surface molecular island. However, although the technique of

controlled carbon monoxide pickup was not used in our study it is instructive to make a comparison between the results of the more commonly used CO tip and an oxygen-down NTCDI. Supplementary Figure 15 shows the calculated force profile for a CO and O:NTCDI tip positioned over the four main sites considered in our study (as shown in Figure 3 of the main text). The initial position of each molecule was set such that each oxygen tip atom is located 0.6nm above the plane of the molecular island. As can be seen from Supplementary Figure 15 the behaviour of the CO and O:NTCDI tip is very similar, supporting our claim that an O:NTCDI tip is capable of comparable resolution to previous studies using the CO method. A key difference observed in our calculations, however, is that the CO tip is significantly more flexible than the NTCDI molecule, evidenced by the behaviour of the force curves at very close approach. This is particularly relevant to the discussion in section V.

Calculated spectra with fixed density

To examine whether atomic and electronic relaxation plays a significant role in determining F(z) we repeated the simulations shown in Figure 3A of the main paper using a simplified approach. In principle the total density is the sum of two terms. 1) the densities of the isolated surface and molecules, and the isolated tip, which can both be obtained by relaxing each of the two systems separately. 2) The density change induced when the surface+molecules and the tip are brought together, which is calculated with the full electronic self-consistency and geometry relaxation:

$$\rho_{total} = \rho_{tip+surface} + \Delta\rho \tag{1}$$

The total energy is a function (in DFT) of the density, so contains terms due to the first and the second terms of the density. Both terms in the density depend on the position of the tip, and hence both contribute to the tip force and eventually to the contrast. However, if the energy due to the first term is more important than that due to the second, then a possible explanation would be that the total density is responsible and its fairly small change due to interaction of the tip with the surface contributes only marginally to contrast. If, however, the energy due to the second term in the density appears to be more important, then the EDD is a better predictor of the contrast in the dynamic force microscopy image.

We therefore ran a series of calculations to test this hypothesis. Specifically, we calculated

the isolated densities of surface + molecule and the tip, and then at each tip height in an F(z) spectrum we calculated only the total energy/force, without allowing the density to further relax. This calculation provided the energy contribution to the first term(isolated density). We then compared the results of this fixed density approach to the fully relaxed case. The results from these calculations are shown in Supplementary Figure 16. From a comparison of the force curves it is clear that there remains a clear separation between the force curves in the repulsive region. Therefore in each case both intermolecular and intramolecular resolution will be observable. In particular we note that the intermolecular features are still visible even when the NTCDI tip is rigidly held in place. The effect of the relaxation of the density, however, is to increase the separation of the force curves in the repulsive region, leading to an improvement in the contrast of the bonds. From these results it appears that the tip-sample force interaction is primarily dominated by the TED, with a small direct contribution from the EDD. As described in the main paper, however, only a percentage of the TED is affected by the repulsive forces that varies with bond type. We believe this observation is crucial to understanding why intra and intermolecular features appear with a similar brightness in experimental measurements.

Simulated images and line profiles

Due to the significantly reduced computational cost of running fixed density and geometry calculations we simulated the full three dimensional force field above a hydrogen bond within the NTCDI island, as shown in Figure 4 of the main paper. The simulated grid was 6×6×2 Å in size using 30×30×10 points corresponding to a grid spacing of 0.2Å. Two dimensional line profiles positioned above single hydrogen and carbon bonds were also calculated using a smaller lateral spacing of 0.1Å (as shown in Figure 4D of the main text). Spectra were calculated over a tip-sample distance range of 5Å to 2Å for the line profiles and 4.5Å to 2.5Å for the full simulated image. Supplementary Figure 17A shows a series of 'slices' taken from the simulated 3D force data. At larger tip-sample distances the image contrast is dominated by short range dispersion interactions which appear as dark depressions, similar to the experimental image in Figure 1D of the main paper. At smaller tip-sample distances the repulsive contribution to the force increases and inter and intra molecular features become apparent as bright protrusions. We note in particular the observed intermolecular contrast in the hydrogen bonding regions. Additional line profiles calculated at multiple tip-sample

separations are shown in Supplementary Figure 17B. Despite the approximations made using a fixed density and geometry the line profile data agrees well with experimental profiles, even though the simulated tip structure is completely fixed in place. The observed oscillations in the simulated images (particularly noticeable at z≥2.8Å) are due to a numerical error associated with the finite grid used in the DFT calculations of the density. A finer SCF grid was tested for a number of points in the simulated force-field and the amplitude of the oscillations reduced significantly. However, this greatly increased the computational cost. Regardless of the grid size used to calculate the density, however, the same overall conclusion is reached: the intermolecular features at the hydrogen-bond positions are observed even when the atomic coordinates and electron density are fixed.

SUPPLEMENTARY REFERENCES

[1] Sweetman, A. *et al.* *Physical review letters* **106**, 136101 (2011).

[2] Majzik, Z. *et al.* *Beilstein Journal of Nanotechnology* **3**, 249–259 (2012).

[3] Weymouth, A., Wutscher, T., Welker, J., Hofmann, T. & Giessibl, F. *Physical review letters* **106**, 226801 (2011).

[4] Rahe, P. *et al.* *Review of Scientific Instruments* **82**, 063704 (2011).

[5] Lantz, M. A. *Science* **291**, 2580–2583 (2001).

[6] Ternes, M. *et al.* *Physical review letters* **106**, 016802 (2011).

[7] Sader, J. E. & Jarvis, S. P. *Applied Physics Letters* **84**, 1801 (2004).

[8] *http://www.cp2k.org* (2013).

[9] VandeVondele, J. *et al.*. *Computer Physics Communications* **167**, 103–128 (2005).

[10] Perdew, J., Burke, K. & Ernzerhof, M. *et al.*. *Physical Review Letters* **77**, 3865 (1996).

[11] Grimme, S., Ehrlich, S. & Goerigk, L. *The Journal of Chemical Physics* **132**, 154104 (2010).

[12] Mohn, F., Schuler, B., Gross, L. & Meyer, G. *Applied Physics Letters* **102**, 073109 (2013).

[13] Gross, L. *et al.* *Science* **337**, 1326–1329 (2012).

[14] Zhang, J. *et al.* *Science* **342**, 611–614 (2013).
